# Determinants and relationships of digital addiction, diet quality, and physical activity in adolescents

**DOI:** 10.3389/fpubh.2025.1654322

**Published:** 2025-09-03

**Authors:** Gizem Helvacı, Fatma Tayhan

**Affiliations:** ^1^Department of Nutrition and Dietetics, Faculty of Health Sciences, Mehmet Akif Ersoy University, Burdur, Türkiye; ^2^Department of Nutrition and Dietetics, Faculty of Health Sciences, Çankırı Karatekin University, Çankırı, Türkiye

**Keywords:** digital addiction, Mediterranean diet, physical activity, adolescent, healthy lifestyle

## Abstract

**Objective:**

Digital addiction, defined as spending excessive time on digital devices and online platforms, is a global problem that particularly affects young people. This form of addiction can lead individuals to a sedentary lifestyle while also increasing the risk of them turning to unhealthy and ready-made foods. This study aimed to determine the prevalence of digital addiction among Turkish adolescents. We also aimed to determine the relationships between digital addiction, adherence to the Mediterranean diet, and physical activity, and to determine the extent to which these variables mutually predict each other.

**Method:**

The data of this cross-sectional study were collected from 400 high school students through a survey. The survey included general information, dietary habits, the Digital Addiction Scale (DAS), the Mediterranean Diet Quality Index (KIDMED), the Leisure Time Exercise Questionnaire (LTEQ), and the Physical Activity Enjoyment Scale (PACES). IBM SPSS 25.0 software was used for data analysis.

**Results:**

Of the students, 23.8% had a digital addiction. Eating during social media use (Beta = 0.158) and night eating habits (Beta = 0.337) positively affected the DAS scores (*p* < 0.05). The KIDMED score (Beta = −0.233) and being physically active (Beta = −0.136) negatively affected the DAS scores (*p* < 0.05). Compliance with the Mediterranean diet was low in 26%, moderate in 46%, and high in 28% of the students. The number of main meals (Beta = 0.254) and father’s education level (Beta = 0.200) positively affected the KIDMED scores (*p* < 0.05). Of the students, 51.5% were active, 32% were moderately active, and 16.5% were sedentary. The PACES scores positively affected the LTEQ scores (Beta = 0.189, *p* < 0.001).

**Conclusion:**

Approximately a quarter of students had digital addiction symptoms. Digital addiction levels tended to decrease as Mediterranean diet adherence and physical activity levels increased. A holistic healthy lifestyle curriculum can be designed to promote healthy eating habits and physical activity among young people, reduce screen time, and increase media literacy. This curriculum, designed for implementation in schools, can support students in engaging in mindful behaviors.

## Introduction

Technology is often beneficial to society, but problems can arise due to its overuse. Long-term and repetitive use of digital devices (such as computers and smartphones) and related activities (such as games and social media) can lead to digital addiction. The term digital addiction covers long-term problems of internet addiction, gaming addiction, and social media addiction (1). There is no consensus yet on the definition or diagnostic criteria of this concept. However, many studies highlight the psychological dependence on digital devices and online platforms (2, 3). It affects both the minds and bodies of children and adolescents. It can lead to visual loss, hearing impairment, and obesity. It can also affect young individuals’ sleeping and eating habits, causing significant damage to their health (4, 5).

Adolescence is a critical period for the acquisition of lifestyle habits that can continue through adulthood. A healthy lifestyle includes quality nutrition, regular physical activity, reduced screen time, avoidance of alcohol and tobacco use, adequate sleep, and positive social interactions (6, 7). It was reported that adolescents generally do not follow healthy lifestyles (7). The courage and curiosity to experiment with different and often unhealthy behaviors increase during this life stage (8). The Mediterranean diet represents a healthy lifestyle approach with nutritional, social, cultural, and environmental characteristics (9). However, compliance with this diet was observed to be low in children and adolescents, including in Mediterranean countries (10). The present study investigated compliance of adolescents to the Mediterranean diet and updated information was provided to the literature.

With the proliferation of the internet, advanced search engines, and social media, public access to information on nutrition and health has become easier (11). Media can stimulate many neural, physiological, and behavioral responses through images and videos of appetizing food (12). Viewing food images was associated with increased secretion of ghrelin, a hormone that increases appetite and calorie intake (13). In adolescents, digital media exposure was reported to increase the consumption of salty, sweet, and fatty foods by affecting their dietary preferences (14) Additionally, problematic use of technology was reported to be associated with meal skipping, appetite changes, irregular eating, snacking behaviors, and poor diet quality (15). Adolescents often exhibit low self-control due to their ongoing cognitive and emotional development (16). Digital addiction further weakens their self-control by overstimulating the reward system and poor self-control is associated with unhealthy food choices and obesity (17, 18). As such, this study examined the relationship between adherence to a Mediterranean diet and digital addiction in adolescents and determined the extent to which these two variables predict each other.

Adolescents’ social lives are increasingly shaped by digital environments, causing virtual interactions to replace physical activity (19). Globally, 8 in 10 adolescents do not engage in sufficient physical activity. Furthermore, a significant portion of adolescents spend more than 2 h a day in front of screens (20). Digital addiction leads to a sedentary lifestyle as it requires constant dependence on a screen (21). However, an optimal physical activity routine and a balanced and adequate diet play an important role in growth and development during adolescence (22). Digital exposure can lead to an unhealthy lifestyle, which, in turn, results in serious problems that negatively affect growth and development (20). Thus, this study also investigated the relationship between physical activity and digital addiction and analyzed the extent to which these two variables predict each other.

Today, since the use of digital devices starts at a younger age, digital addiction has become a global problem especially affecting young people (23). There are many studies in the literature in which a specific subtype of digital addiction, e.g., internet addiction and gaming addiction, has been addressed. However, digital technology is rapidly advancing and constantly changing. There are relatively few studies in which the overuse of digital devices, digital technologies, and digital platforms has been holistically evaluated. The assessment tool used in this study will help determine adolescents’ overexposure to technology in all its forms. We aimed to determine the relationship between digital addiction and diet quality, eating habits, exercise level, and enjoyment of exercise and expand the knowledge in this field.

## Method

The study followed a cross-sectional research design and was conducted with 400 adolescent students studying in schools affiliated to the Ministry of National Education in Turkey. Participants were recruited used a convenience sampling method, which was based on accessibility and willingness to participate. Individuals aged between 11 and 19 years old who were using technological tools (computer, laptop, and phone) and who volunteered to participate in the study were included. Individuals with a physical or mental disability that prevented them from reading and understanding the survey questions were excluded from the study.

The research data were collected using a survey during face-to-face interviews at the schools. The individuals who agreed to participate in the study completed the survey, which included general information, eating habits, the Digital Addiction Scale (DAS), the Mediterranean Diet Quality Index (KIDMED), the Leisure Time Exercise Questionnaire (LTEQ), and the Physical Activity Enjoyment Scale (PACES). Before the survey was applied, the participants and their families were informed about the study and asked to complete the consent form.

### Digital addiction scale for teenagers (DAS)

The DAS scale was developed by Seema et al. (24) to measure the digital addiction level of young individuals (24). The Turkish validity and reliability study was conducted by Çelik et al. in 2023 (25). The Turkish version of the DAS for Teenagers consists of 10 items. The scale has a 5-point Likert-type rating (never-always). This scale is a valid and reliable measurement tool for determining the digital addiction levels of young individuals aged between 11 and 19 years old or studying in middle and high school. The scale has no reverse items. The higher the scale score (between 10 and 50 points), the higher the risk of digital addiction (25).

### Mediterranean diet quality index (KIDMED)

The KIDMED scale was developed by Serra Majem et al. in 2004 to determine the appropriateness of individuals’ eating habits to the Mediterranean diet (26). The Turkish validity and reliability study was conducted by Şahingöz et al. in 2019 (27). The scale consists of 16 items. Items 6, 12, 14, and 16 are scored −1 and the remaining 12 items are scored +1. A score of 3 or below indicates low diet quality, a score between 4 and 7 indicates moderate diet quality, and a score of 8 or more indicates high diet quality (27).

### Leisure time exercise questionnaire (LTEQ)

The LTEQ was developed by Godin and Shephard (28) to measure the exercise activity of individuals during leisure time (28). The Turkish validity and reliability study for adolescents was conducted by Lapa et al. (29). The questionnaire includes questions about physical activity performed for at least 15 min during leisure time in the last week. Depending on the frequency of the activity, heavy activities are multiplied by a score of 9, moderate activities by 5, and mild activities by 3. The weekly leisure time activity score is calculated with the formula (9 x severe intensity) + (5 x moderate intensity) + (3 x mild intensity). Accordingly, an individual’s activity during leisure time in general is assessed. The score is classified at three levels: 24 and above as “active,” 14 to 23 as “moderately active,” and 13 or below as “not active enough” (29).

### Physical activity enjoyment scale (PACES)

The PACES was developed by Mullen et al. (30) to assess expected and perceived positive emotions regarding physical activity (30). The Turkish validity and reliability study was conducted by Özkurt et al. (31). The scale consists of 8 items that are scored using a 7-point Likert-type scale. The scale is scored between 8 and 56 and a high score indicates a high level of physical activity enjoyment (31).

### Statistical analysis

Data analysis was conducted with the IBM Statistical tool for Social Sciences (SPSS) 25.0 tool. Descriptive statistics included mean, standard deviation, number, and percentage values. The independent samples t-test and the Pearson chi-square test were used to compare continuous and categorical variables, respectively. The relationships between the scale scores were determined through correlation and regression analysis.

The discriminative power of the DAS scores, which is used to determine a participant’s digital addiction status, was evaluated with ROC analysis. As a result of the analysis, the area under the ROC curve (AUC value) was 0.921 (95% CI: 0.884–0.958). According to this value, the discriminative power of the scale was high in predicting digital addiction in the participants. The most appropriate cut-off point to discriminate between addicted and non-addicted individuals according to the DAS score was determined to be 28.5.

## Results

The distribution of the demographic characteristics of the participants according to gender is given in Table 1. A total of 400 students participated in the study. Of these students, 57.2% were female (n = 229) and 42.8% (n = 171) were male. The mean age of the participants was 14.8 ± 1.0 years. In terms of the educational status of the parents of the participants, the rate of primary or middle school graduates among the mothers (41.3%) and the rate of high school graduates among the fathers (50.4%) were higher. The rate of male students who regularly exercised (47.4%) was higher than female students who regularly exercised (28.8%; *p* < 0.001). The purpose of participation in exercise did not differ statistically according to gender, and the rate of those who exercised to compete and have fun (43.5%) was higher. Of the students, 39.5% exercised to be healthy and 17% exercised to lose weight. The rate of smoking (5.8%) and alcohol consumption (5.3%) was higher in male students than in female students (*p* = 0.027 and *p* = 0.003, respectively).

**Table 1:** Distribution of demographic characteristics of students by gender.

Demographic characteristics	Female (*n* = 229)		Male (*n* = 171)		Total (*n* = 400)		*p*-value
	*n*	%	*n*	%	*n*	%	
Age (year)							
14	130	56.8	107	62.6	237	59.2	0.513^a^
15	34	14.8	24	14.0	58	14.5	
16	43	18.8	23	13.5	66	16.5	
17	22	9.6	17	9.9	39	9.8	
Mean (X̅ ±SD)	14.8 ± 1.1		14.7 ± 1.0		14.8 ± 1.0		0.323^b^
Mother’s education level							
Illiterate	10	4.4	1	0.6	11	2.8	**0.005** ^**a**^
Primary-middle school	92	40.2	73	42.7	165	41.3	
High school	89	38.9	50	29.2	139	34.8	
University	30	13.1	42	24.6	72	18.0	
Graduate degree	8	3.5	5	2.9	13	3.3	
Father’s education level							
Illiterate	5	2.2	2	1.2	7	1.8	0.405^a^
Primary-middle school	48	21.0	32	18.7	80	20.0	
High school	110	48.0	92	53.8	202	50.4	
University	55	24.0	32	18.7	87	21.8	
Graduate degree	11	4.8	13	7.6	24	6.0	
Regular exercise status							
Yes	66	28.8	81	47.4	147	36.8	**<0.001** ^**a**^
No	163	71.2	90	52.6	253	63.2	
Purpose of exercise							
To be healthy	93	40.6	65	38.0	158	39.5	0.215^a^
To lose weight	44	19.2	24	14.0	68	17.0	
To compete-have fun	92	40.2	82	48.0	174	43.5	
Physical activity status							
Yes	181	79.0	145	84.8	326	81.5	0.142^a^
No	48	21.0	26	15.2	74	18.5	
Smoking status							
Yes	4	1.7	10	5.8	14	3.5	**0.027** ^**c**^
No	225	98.3	161	94.2	386	96.5	
Alcohol consumption status							
Yes	1	0.4	9	5.3	10	2.5	**0.003** ^**c**^
No	228	99.6	162	94.7	390	97.5	

Significant *p* values are indicated in bold.

a Pearson Chi-Square Test.

b Independent Samples t Test.

c Fisher’s exact test.

The distribution of the students’ eating habits and scale scores according to their digital addiction status is given in Table 2. The rate of skipping main meals was 40% in the students with a digital addiction and 21.6% in those without a digital addiction (*p* < 0.001). Breakfast was the most frequently skipped meal in both groups, and the rate of skipping breakfast was higher among the students with a digital addiction (82.1%; *p* = 0.017). Night eating habits (63.2%; *p* < 0.001), fast eating behavior (22.1%; *p* = 0.006), and eating during social media use (93.7%; *p* < 0.001) were also higher in the students with a digital addiction compared to those without a digital addiction. The rate of students with low diet quality was statistically significantly higher in the students with a digital addiction (48.4%) compared to the students without a digital addiction (19.3%; *p* < 0.001). According to digital addiction status, the mean total PACES and LTEQ scores were similar between the groups (*p* > 0.05). However, in the classification according to LTEQ scores, the rate of physically inactive students was higher among the students with a digital addiction (23.2%; *p* < 0.001). The mean body mass index (BMI) of the students was 20.7 ± 3.6 kg/m^2^ and did not differ statistically according to digital addiction status (*p* > 0.05).

**Table 2:** Distribution of eating habits and scale scores of students according to their digital addiction status.

Variables	Digital addiction						
	Yes (*n* = 95)		No (*n* = 305)		Total (*n* = 400)		*p*-value
	*n*	%	*n*	%	*n*	%	
Skipping main meals							
Yes	38	40.0	66	21.6	104	26.0	**<0.001** ^**a**^
No	57	60.0	239	78.4	296	74.0	
Number of main meals (X̅ ±SD)	2.7 ± 0.5		2.8 ± 0.4		2.8 ± 0.4		**<0.001** ^**b**^
Skipped main meal							
Breakfast	32	82.1	102	63.7	134	67.4	**0.017** ^**a**^
Lunch	4	10.2	52	32.5	56	28.1	
Dinner	3	7.7	6	3.8	9	4.5	
Skipping snacks							
Yes	42	44.2	157	51.5	199	49.8	0.216^a^
No	53	55.8	148	48.5	201	50.2	
Number of snacks (X̅ ±SD)	1.5 ± 0.9		1.6 ± 0.9		1.5 ± 0.9		0.354^b^
Thinking that you have an adequate and balanced diet							
Yes	39	41.1	164	53.8	203	50.7	**0.010** ^**a**^
No	22	23.2	35	11.5	57	14.2	
I do not know	34	35.8	106	34.5	140	35.0	
Night eating habit							
Yes	60	63.2	75	24.6	135	33.8	**<0.001** ^**a**^
No	35	36.8	230	75.4	265	66.3	
Eating speed							
Slow	11	11.6	28	9.2	39	9.8	**0.006** ^**a**^
Normal	63	66.3	246	80.7	309	77.2	
Fast	21	22.1	31	10.1	52	13.0	
Social media eating							
Yes	89	93.7	228	74.8	317	79.3	**<0.001** ^**a**^
No	6	6.3	77	25.2	83	20.8	
KIDMED							
Low	46	48.4	59	19.3	105	26.3	**<0.001** ^**a**^
Moderate	36	37.9	149	48.9	185	46.3	
High	13	13.7	97	31.8	110	27.5	
PACES							
Total score (X̅ ±SD)	41.2 ± 12.9		39.4 ± 12.3		39.8 ± 12.4		0.211^b^
LTEQ							
Total score (X̅ ±SD)	33.5 ± 24.8		33.2 ± 25.9		33.3 ± 25.7		0.920^b^
Not active enough	22	23.2	44	14.4	66	16.5	**<0.001** ^**a**^
Moderately active	16	16.8	112	36.7	128	32.0	
Active	57	60.0	149	48.9	206	51.5	
BMI (kg/m^2^) (X̅ ±SD)	20.8 ± 4.4		20.7 ± 3.3		20.7 ± 3.6		0.892^b^

PACES: Physical Activity Enjoyment Scale, KIDMED: Mediterranean Diet Quality Index for Children and Adolescents, LTEQ: Leisure Time Exercise Questionnaire, BMI: Body Mass Index. Significant *p* values are indicated in bold.

a Pearson Chi-Square Test.

b Independent Samples T Test.

**Figure 1:**
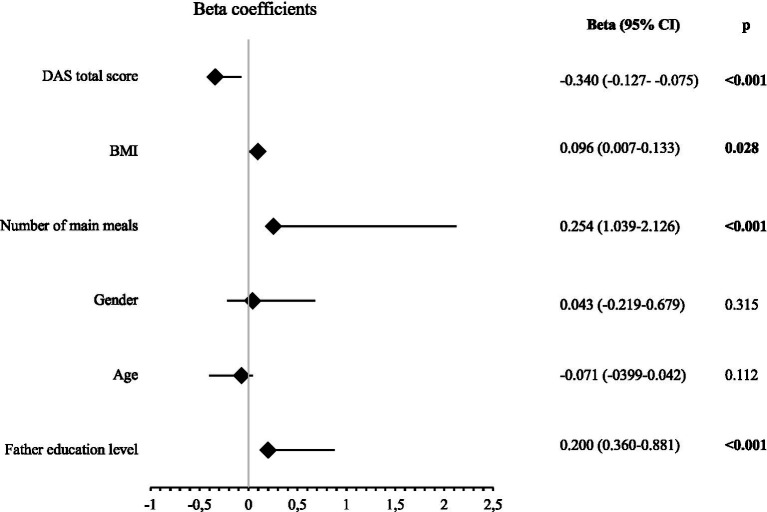
Forest Plot of linear regression analysis for KIDMED total score (DAS: digital addiction scale, BMI: body mass index, significant *p* values are indicated in bold). Gender (coded: 0 = Female, 1 = Male).

Figure 1 shows the factors affecting the KIDMED scores in a forest plot. There was a statistically significant negative correlation between the DAS scores and the KIDMED scores (Beta = −0.340, *p* < 0.001). The total KIDMED scores of the students increased as the education level of their fathers increased (Beta = 0.200, *p* < 0.001). The increase in BMI of the students positively affected their total KIDMED scores (Beta = 0.096, *p* = 0.028). An increase in the number of main meals positively affected the KIDMED scores (Beta = 0.254, *p* < 0.001). Gender and age had no effect on the KIDMED scores (*p* > 0.05).

**Figure 2:**
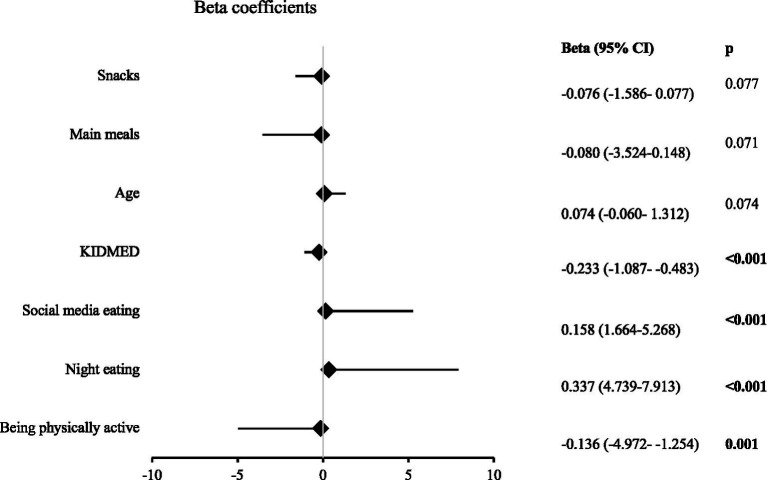
Results of linear regression analysis for the total DAS score [DAS: digital addiction scale, significant *p* values are indicated in bold. Social media eating (coded: 0 = No, 1 = Yes). Night eating (coded: 0 = No, 1 = Yes). Being physically active (coded: 0 = No, 1 = Yes)].

Figure 2 shows the factors that affected the DAS scores. Eating during social media use (Beta = 0.158, *p* < 0.001) and night eating habits (Beta = 0.337, p < 0.001) positively affected the DAS scores. The DAS scores decreased as the KIDMED scores increased (Beta = −0.233, *p* < 0.001). Being physically active negatively affected the DAS scores (Beta = −0.136, *p* = 0.001). Age, number of main meals, and number of snacks had no effect on the DAS scores (*p* > 0.05).

**Figure 3:**
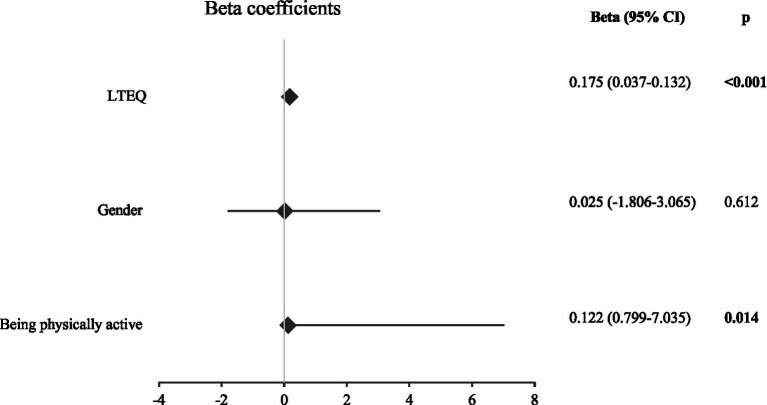
Results of linear regression analysis for the total PACES score [LTEQ: leisure time exercise questionnaire, significant *p* values are indicated in bold. Gender (coded: 0 = Female, 1 = Male). Being physically active (coded: 0 = No, 1 = Yes)].

Figure 3 shows the factors that affected the PACES scores. Being physically active had a positive and significant effect (Beta = 0.122, *p* = 0.014). The LTEQ scores also positively affected physical activity enjoyment (Beta = 0.175, *p* < 0.001). Gender was not among the affecting factors (Beta = 0.025, *p* = 0.612).

**Figure 4:**
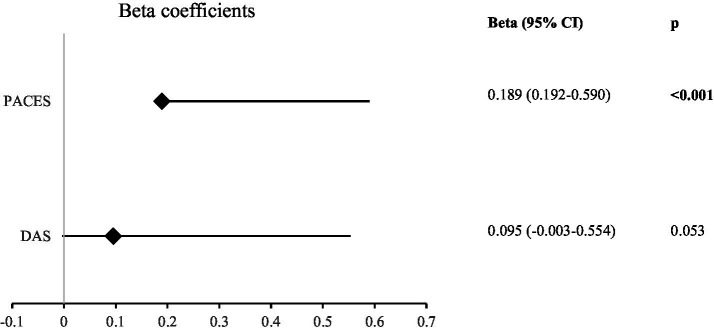
Results of linear regression analysis for the total LTEQ score (LTEQ: leisure time exercise questionnaire, DAS: digital addiction scale, PACES: physical activity enjoyment scale, significant *p* values are indicated in bold).

When the factors affecting the level of leisure time exercise (LTEQ) were analyzed, physical activity enjoyment (Beta = 0.189, *p* < 0.001) had a positive effect. The effect of the DAS scores (Beta = 0.095, *p* = 0.053) on the LTEQ scores was statistically insignificant (*p* > 0.05; Figure 4).

Table 3 shows the correlations between the scale scores and some variables. There was a negative correlation between the students’ DAS and KIDMED scores (r = −0.419, *p* < 0.001). There was a significant negative correlation between the DAS scores and the number of main meals consumed (r = −0.238, *p* < 0.001). The DAS scores of the students increased as their age increased (r = 0.182, *p* < 0.001). There was a positive correlation between the students’ compliance with the Mediterranean diet and their number of main meals (r = 0.354, *p* < 0.001). In addition, as compliance with the Mediterranean diet increased, the number of snacks increased (r = 0.227, *p* < 0.001). The students’ compliance with the Mediterranean diet increased as the educational level of their fathers increased (r = 0.211, *p* < 0.001). The students’ KIDMED scores, number of main meals, and LTEQ scores decreased as their age increased (*p* < 0.05).

**Table 3:** Correlations between scale scores and some variables.

Variables		1. DAS	2. PACES	3. KIDMED	4. LTEQ	5. BMI	6. Number of main meals	7. Number of snacks	8. Mother education level	9. Father education level	10. Age
1. DAS	r	1									
	*p*										
2. PACES	r	0.072	1								
	*p*	0.149									
3. KIDMED	r	−0.419^**^	0.024	1							
	*p*	<0.001	0.627								
4. LTEQ	r	0.109^*^	0.196^**^	0.038	1						
	*p*	0.029	<0.001	0.451							
5. BMI	r	−0.010	0.049	0.076	−0.089	1					
	*p*	0.836	0.331	0.131	0.075						
6. Number of main meals	r	−0.238^**^	0.011	0.354^**^	−0.040	−0.074	1				
	*p*	<0.001	0.820	<0.001	0.420	0.139					
7. Number of snacks	r	−0.146^**^	0.017	0.227^**^	0.039	−0.008	0.272^**^	1			
	*p*	0.003	0.734	<0.001	0.440	0.879	<0.001				
8. Mother education level	r	0.015	0.054	0.068	−0.086	0.072	0.065	−0.010	1		
	*p*	0.760	0.286	0.175	0.085	0.152	0.192	0.842			
9. Father education level	r	−0.003	0.053	0.211^**^	−0.123^*^	0.035	0.052	0.026	0.371^**^	1	
	*p*	0.952	0.292	<0.001	0.014	0.480	0.301	0.600	<0.001		
10. Age	r	0.182^**^	−0.056	−0.134^**^	−0.128^*^	0.203^**^	−0.153^**^	−0.037	0.087	0.100^*^	1
	*p*	<0.001	0.262	0.007	0.010	<0.001	0.002	0.455	0.081	0.045	

*p < 0,05, **p < 0,001, DAS: Digital Addiction Scale, PACES: Physical Activity Enjoyment Scale, KIDMED: Mediterranean Diet Quality Index for Children and Adolescents, LTEQ: Leisure Time Exercise Questionnaire, significant *p* values are indicated in bold. BMI: Body Mass Index.

## Discussion

Digital addiction has emerged with the advancement of technology and has steadily spread globally. Moreover, the age of digital addicts has tended to decrease over time (32). Adolescents are more vulnerable to digital addiction due to various biological, psychological, and social factors (33, 34). In the present study, 23.8% of the high school students were found to have a digital addiction. In a meta-analysis study, a quarter of the general population was reported to suffer from at least one subtype of digital addiction (35). The prevalence reported for adolescents is wide and can reach higher levels than that of the general population (3–62%) (36–38). The evolving nature of digital engagement among young people and the methods used to determine their addiction status may explain the variability in reported prevalences.

Compliance with the Mediterranean diet, which is a health-promoting dietary pattern, tends to decline among adolescents (39). In our study, 26% of the students had low compliance with the Mediterranean diet, 46% had moderate compliance, and 28% had high compliance. In previous studies, the rate of moderate compliance constituted the majority, but the rate of ideal compliance was higher than or similar to the rate found in our study (40, 41). Despite living in a country on the Mediterranean coast, there is a need to improve Turkish adolescents’ commitment to a healthy dietary pattern. Several strategies for education and access to healthy foods should be developed and implemented to increase the rate of students with high levels of compliance with the Mediterranean diet.

In the present study, the main determinants of the students’ compliance with the Mediterranean diet were their fathers’ education level, number of main meals, BMI, and level of digital addiction. The children of fathers with higher education levels had better diet quality. Likewise, parental education level was reported to play an important role in shaping their children’s dietary behaviors (42). The students who consumed their main meals regularly were found to have high compliance with the Mediterranean diet. Similar results were obtained in the study conducted by Alim et al. (43), which showed that an increase in the frequency of meals was positively associated with dietary diversity and compliance with the Mediterranean diet (43). In our study, the students’ level of digital addiction was negatively correlated with their compliance with the Mediterranean diet. In previous research, it has been confirmed that the widespread use of technology and digital interaction among adolescents have a significant negative impact on food preferences and diet quality (44, 45).

Digital addiction is a new type of addiction that shares similar characteristics with other addictive behaviors (46). The World Health Organization emphasized the potential for digital devices to disrupt daily activities and well-being when used excessively and has recognized digital addiction as a mental health problem (47). Due to its prevalence and harmful effects, it is important to identify factors that predict digital addiction. In the present study, eating during social media use and night eating behaviors were among the determinants of digital addiction. Previously, eating in front of a screen was reported to be common among adolescents (48). This behavior can lead to increased screen time and time spent on social media, resulting in symptoms of digital addiction. Eating late at night can lead to delayed sleep and increased preoccupation with digital devices (49). According to the findings, other determinants of digital addiction were compliance with the Mediterranean diet and being physically active, which negatively affected digital addiction. The Mediterranean diet is known for its potential to promote a healthy lifestyle and social interactions (50). This characteristic may contribute to reducing the time spent using digital devices. It has been previously reported that in children, the types of digital activities (playing digital games, watching television, and watching streaming platforms) are associated with lower adherence to the Mediterranean diet (51). Other studies have reported that as social media addiction increases in young people, adherence to the Mediterranean diet decreases and the risk of eating disorders increases (52, 53). Another study reported that children’s use of television or gaming computers during off-school hours partially displaced the time they should have spent eating. Poor eating habits, including fast eating, skipping meals, snacking, and the disruption of family meal planning, may undermine adherence to the Mediterranean diet (54). Furthermore, consistent with our findings, many studies have reported that participation in regular physical activity protects against digital addiction in adolescents (55, 56). Physical activity can increase self-esteem, encourage socialization, and reduce the use of digital devices (57).

The current study found a bidirectional relationship between physical activity level and the enjoyment of physical activity. The fact that these variables mutually predict each other is a remarkable finding in terms of the continuity of physical activity behavior. Enjoyment from physical activity is important for the physical, psychological, and social development of adolescents. Consistent with our findings, it is reported to support participation in more physical activity (58–60). The results of this study demonstrated the importance of focusing on the emotional satisfaction that is derived from this behavior in interventions aimed at increasing physical activity. Additionally, exercise improves mood by modulating hormone and neurotransmitter levels (61). As individuals gain both physical and psychological benefits from exercise, they may be more likely to continue this behavior. Even if exercise participation is initially reluctant, feelings of enjoyment may develop over time. This positive cycle, reinforced by hormonal responses, may regulate voluntary participation in physical activity (62).

## Strengths and limitations

This study’s strength is its holistic perspective, assessing the interrelationships between digital addiction, Mediterranean diet adherence, and exercise in adolescents. Additionally, using trusted and tested measurement tools (DAS, KIDMED, LTEQ, and PACES), which have been adjusted for Turkish teenagers, improves the quality and trustworthiness of the study’s results. However, the study also has several limitations. Firstly, due to its cross-sectional design, a causal relationship cannot be established between the variables that were examined. Longitudinal studies should be planned to explore the causality between digital addiction, healthy eating, and physical activity over time. Secondly, the use of convenience sampling may limit the representativeness of the sample, which could affect the generalizability of the findings. Selection bias is also a concern, as participants with similar characteristics may be overrepresented in this study. Thirdly, the study used a self-administered survey for the adolescents who participated, which may lead to recall and social desirability bias. Fourthly, although the sample represents the population, the sample size was relatively small. Further research on the subject, including larger and multicenter samples, is recommended. Finally, although the study utilized validated scales, the absence of qualitative data limits the depth of understanding regarding the psychosocial dimensions of digital addiction. Future research could be improved by using qualitative methods, including interviews, to better understand the complex experiences and situations that contribute to digital addiction. This approach would enhance the comprehensiveness of the findings.

## Conclusion

Digital addiction symptoms were identified in about a quarter of the surveyed high school students. It is important to examine risk and protective factors to protect the young generation from the harms of the digital age. According to our results, eating during social media use and night eating habits are risk factors for digital addiction, while compliance with the Mediterranean diet and physical activity are preventive factors.

This study, which also provides information on diet and physical activity, gives an idea about the lifestyle of high school students living in Turkey. A large majority of the students had a moderate level of compliance with the Mediterranean diet. Parents and educators should encourage students to adopt the Mediterranean diet as a healthy lifestyle choice. Families can limit unhealthy snacks at home and involve children in healthy meal preparation. Educators can model healthy eating behaviors through school activities. Policymakers can support adherence to the Mediterranean diet by facilitating access to healthy foods and allocating resources to school-based nutrition programs. In this study, approximately half of the students were physically active. Physical activity enjoyment positively affected the activity level of the students. Providing opportunities for students to identify which type of physical activity they enjoy and education that emphasizes the importance of physical activity may be some ways to promote sustainable physical activity habits.

Additionally, families, educators, and policymakers can take measures to combat digital addiction. Technology-free areas and time periods can be created to increase face-to-face communication in schools and homes. Additionally, establishing rules to limit device use may prove effective. Policymakers can impose stricter regulations on games and applications that target young people. A holistic healthy lifestyle curriculum can be designed to promote healthy eating habits and physical activity among young people, to reduce screen time, and to increase media literacy. This curriculum, which is designed for implementation in schools, could support students in engaging in mindful behaviors. To guide families, educators and policy makers, further studies are required to explore all lifestyle habits in more detail and to identify the effects of digital addiction.

## Data Availability

The raw data supporting the conclusions of this article will be made available by the authors, without undue reservation.
